# West Nile Virus Surveillance in 2013 via Mosquito Screening in Northern Italy and the Influence of Weather on Virus Circulation

**DOI:** 10.1371/journal.pone.0140915

**Published:** 2015-10-21

**Authors:** Mattia Calzolari, Alessandra Pautasso, Fabrizio Montarsi, Alessandro Albieri, Romeo Bellini, Paolo Bonilauri, Francesco Defilippo, Davide Lelli, Ana Moreno, Mario Chiari, Marco Tamba, Mariagrazia Zanoni, Giorgio Varisco, Silvia Bertolini, Paola Modesto, Maria Cristina Radaelli, Barbara Iulini, Marino Prearo, Silvia Ravagnan, Stefania Cazzin, Paolo Mulatti, Isabella Monne, Lebana Bonfanti, Stefano Marangon, Maria Goffredo, Giovanni Savini, Simone Martini, Andrea Mosca, Marco Farioli, Laura Gemma Brenzoni, Manlio Palei, Francesca Russo, Silvano Natalini, Paola Angelini, Cristina Casalone, Michele Dottori, Gioia Capelli

**Affiliations:** 1 Istituto Zooprofilattico Sperimentale della Lombardia e dell’Emilia Romagna, Brescia, Italy; 2 Istituto Zooprofilattico del Piemonte Liguria e Valle d'Aosta, Torino, Italy; 3 Istituto Zooprofilattico Sperimentale delle Venezie, Leganro (PD), Italy; 4 Centro Agricoltura Ambiente “G.Nicoli,” Crevalcore (BO), Italy; 5 Istituto Zooprofilattico Spermentale dell’Abruzzo e Molise, Teramo, Italy; 6 Entostudio snc, Brugine (PD), Italy; 7 Istituto per le Piante da Legno e l'Ambiente S.p.A., Torino, Italy; 8 Regione Lombardia, Milano, Italy; 9 Regione Friuli Venezia-Giulia, Trieste, Italy; 10 Regione Veneto, Venezia, Italy; 11 Regione Emilia-Romagna, Bologna, Italy; University of California Davis, UNITED STATES

## Abstract

West Nile virus (WNV) is a recently re-emerged health problem in Europe. In Italy, an increasing number of outbreaks of West Nile disease, with occurrences of human cases, have been reported since 2008. This is particularly true in northern Italy, where entomological surveillance systems have been implemented at a regional level. The aim of this study was to use, for the first time, all the entomological data collected in the five regions undergoing surveillance for WNV in northern Italy to characterize the viral circulation (at a spatial and temporal scale), identify potential mosquito vectors, and specify relationships between virus circulation and meteorological conditions. In 2013, 286 sites covering the entire Pianura Padana area were monitored. A total of 757,461 mosquitoes were sampled. Of these, 562,079 were tested by real-time PCR in 9,268 pools, of which 180 (1.9%) were positive for WNV. The largest part of the detected WNV sequences belonged to lineage II, demonstrating that, unlike those in the past, the 2013 outbreak was mainly sustained by this WNV lineage. This surveillance also detected the Usutu virus, a WNV-related flavivirus, in 241 (2.6%) pools. The WNV surveillance systems precisely identified the area affected by the virus and detected the viral circulation approximately two weeks before the occurrence of onset of human cases. Ninety percent of the sampled mosquitoes were *Culex pipiens*, and 178/180 WNV-positive pools were composed of only this species, suggesting this mosquito is the main WNV vector in northern Italy. A significantly higher abundance of the vector was recorded in the WNV circulation area, which was characterized by warmer and less rainy conditions and greater evapotranspiration compared to the rest of the Pianura Padana, suggesting that areas exposed to these conditions are more suitable for WNV circulation. This observation highlights warmer and less rainy conditions as factors able to enhance WNV circulation and cause virus spillover outside the sylvatic cycle.

## Introduction

West Nile virus (WNV) (Flaviviridae: *Flavivirus*) is an arbovirus circulating among mosquitoes, which serve as the vectors, and wild birds, which serve as the main reservoir hosts. WNV is characterized by a complex pattern of circulation, involving different vector and reservoir species in different areas and ecological conditions, depending particularly on mosquito and bird species richness and abundance [1, 2]. The plasticity and adaptability of this virus to different habitats has allowed its worldwide expansion and makes its monitoring and modeling a difficult task.

WNV raises public health and veterinary concerns for its ability to infect horses and humans, which are considered dead-end hosts due to their inability to develop a sufficient viremia to infect mosquitoes [2, 3]. Approximately 8% of infected horses develop neurologic symptoms, mainly encephalomyelitis with ataxia [4], whereas infected humans can develop symptoms in approximately 20–40% of infections. The symptoms in humans may range from mild influenza-like syndrome (West Nile fever, WNF) to severe West Nile neuroinvasive disease (WNND), characterized by meningitis, encephalitis, and acute flaccid paralysis, in approximately 1% of infected humans [5]. In addition to the direct risk to human health, asymptomatic blood donors represent a recognized problem for the safety of blood transfusions in affected areas [2, 5].

WNV is recognized as a threat in Europe, with 826 human cases of WNF/WNND reported in the period 2010–2013 in the European Union. Of these, 226 cases occurred in 2013 and 69 cases (30.5%) were recorded in Italy [6]. WNV was first detected in Italy in 1998 in the Tuscany region in horses, with no human cases recorded [7]. Then, after ten years, WNV reappeared northward in Pianura Padana, the largest plain of Italy [8], affecting the Emilia-Romagna, Veneto, and Lombardia regions, and causing at least 8 human cases of WNND [9]. Different strains of the virus were detected in different areas of Pianura Padana every subsequent year prior to 2013 [10–12].

Infection in the dead-end hosts is not important to the integrity of the viral cycle [13]; consequently, human and equine West Nile disease cases appear after the occurrence of WNV amplification between vectors and vertebrate competent hosts. Therefore, environmental surveillance, particularly surveillance based on mosquito and wild bird sampling, can provide early detection of the virus circulation before the onset of the disease in humans and horses [14, 15]. For this reason, as a complement to the activities supported by the Ministry of Health at the national level, more comprehensive surveillance programs, including mosquito monitoring, have been conducted in Pianura Padana and bordering areas at the regional level beginning since 2008 [10, 16, 17]. These surveillance systems have given encouraging results in terms of the sensitivity and early detection of the virus [18–22] and have also allowed the detection of the widely circulating Usutu virus (USUV) [23], a WNV-related flavivirus that is pathogenic in birds and rarely pathogenic in immunocompromised humans [24, 25] but serologically detected in healthy people in the surveyed areas [26, 27].

For the first time, in 2013, five neighboring regions of northern Italy organized comparable programs of entomological surveillance, allowing the monitoring of the entire Pianura Padana territory (approximately 46,000 km^2^). The data obtained by this surveillance are described and analyzed in this article with the aim of characterizing the viral circulation (at a spatial and temporal scale) and of identifying relationships among virus circulation, meteorological conditions and mosquito abundance.

## Results

**Fig 1:**
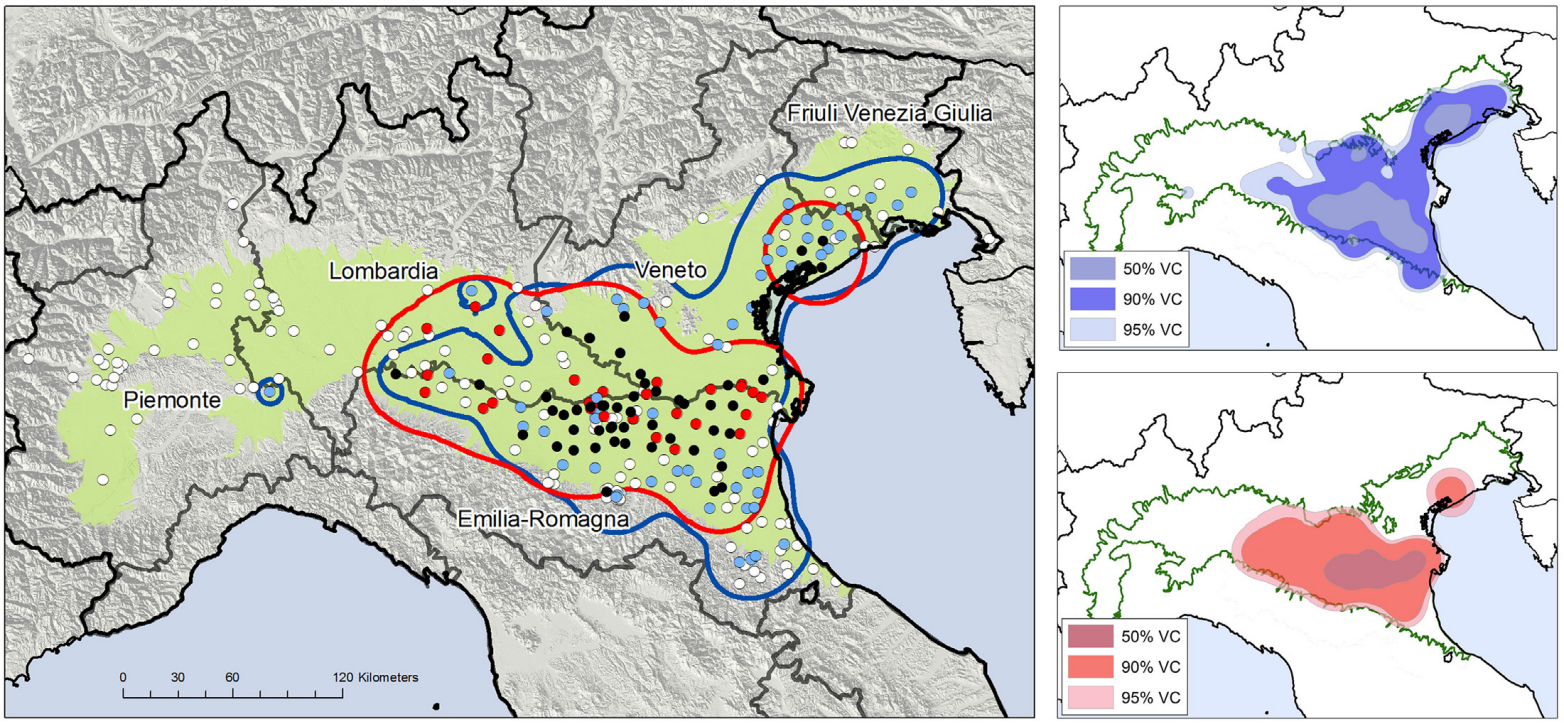
WNV and USUV detections. Surveyed area with sampling stations and locations of virus detection (red, WNV-positive; azure, USUV-positive; black, WNV- and USUV-positive) and borders of the WNV (red line) and USUV (azure line) circulation areas. On the right: volume contour (VC) of the WNV-circulation area (red) and of the USUV-circulation area (blue) estimated by kernel density estimation (KDE). The plain area and plain limit are reported in green.

A total of 286 sites were monitored in 2013. Of these, 185 were sampled on at least six occasions, with a maximum interval of two weeks, between May and October (88 in Emilia-Romagna, 51 in Veneto, 31 in Piemonte, 11 in Friuli Venezia-Giulia, and 4 in Lombardia). A further 101 sites were monitored fewer than six times during the surveillance season (Fig 1, S1 Fig).

A total of 757,461 mosquitoes belonging to 21 species were collected. More than 90% were identified as *Culex pipiens* L., followed by *Aedes (Ochlerotatus) caspius* (Pallas) (5.8%) and *Aedes vexans* (Meigen) (1.9%) specimens (Table 1, S1 Table). Mosquitoes recorded at an abundance >0.2% but <1% were *Culex modestus* Ficalbi, the species of the *Anopheles maculipennis* complex and the invasive *Aedes (Stegomyia) albopictus* (Skuse). The number of collected mosquitoes varied depending on the period, area of sampling (S2 Fig) and weather conditions. The median monthly averages of *Cx*. *pipiens* specimens collected per site in the area of the plain (including stations checked more than six times) were 564 specimens in June (132 sites), 390 in July (152 sites), 179 in August (158 sites), and 64 in September (158 sites). The most abundant collection (19,460 mosquitoes) was on June 26 from a single site in Ferrara Province, near the Adriatic Sea.

**Table 1:** Collected specimens per species (N), number of tested mosquitoes (Nt) and tested pools (Np), and WNV- and USUV-positive pools.

Species	N (%)	Nt	Np	Wnv+	Usuv+
*Ae*. *cinereus/geminus*	111(<0.1)	95	6		
*Ae*. *cantans*	171(<0.1)	74	26		
*Ae*. *caspius*	44,108(5.8)	31,842	844	1	2
*Ae*. *detritus*	693(<0.1)	117	19		
*Ae*. *albopictus*	4,659(0.6)	2,648	463		
*Ae*. *geniculatus*	214(<0.1)	130	9		
*Ae*. *koreicus*	26(<0.1)	6	5		
*Ae*. *vexans*	14,436(1.8)	11,694	257		
*An*. *claviger/petragnani*	16(<0.1)	10	8		
*An*. *maculipennis s*.*l*.	5,445(0.7)	2,704	156		
*An*. *plumbeus*	55(<0.1)	35	12		
*Cq*. *richiardii*	335(<0.1)	247	39		
*Cs*. *annulata*	246(<0.1)	203	82		
*Cx*. *modestus*	2,231(0.3)	1,450	75	1	1
*Cx*. *pipiens*	684,660 (90)	510,773	7,256	178	238
*Cx*. *territans*	42(<0.1)	39	5		
Other species^1^	14(<0.1)	13	6		
Total	757,461	562,079	9,268	180	241

Genera abbreviations, *Ae*., *Aedes*; *An*., *Anopheles; Cq*., *Coquillettidia; Cs*., *Culiseta*; *Cx*., *Culex*.

^1^ *Ae*. *(Och*.*) berlandi* (1), *Ae*. *(Och*.*) sticticus* (3), *Ae*. *(Och*.*) flavescens* (1), *Aedes spp*. (7), *Cs*. *subchorea* (1), *Cx*. *hortensis* (1).

**Fig 2:**
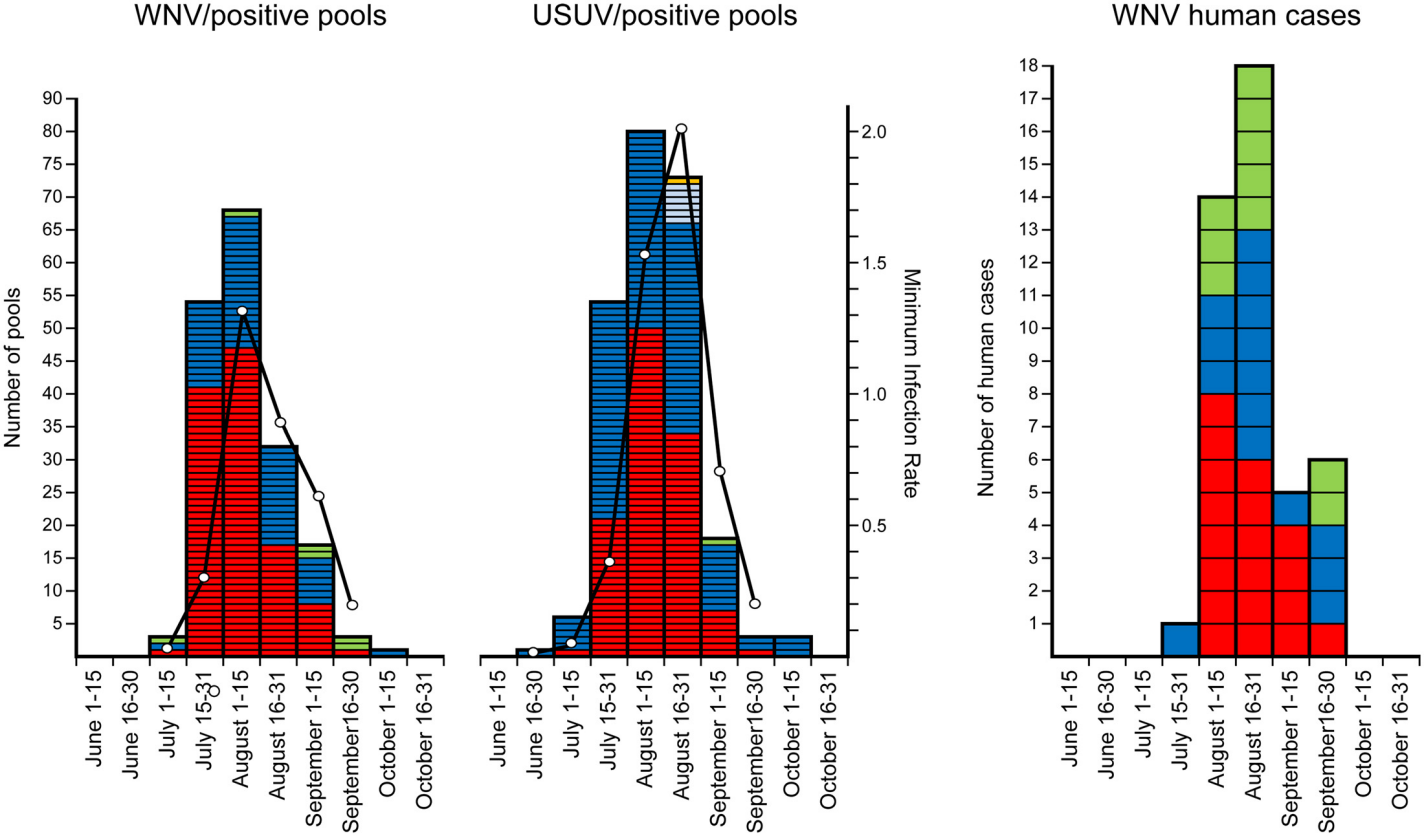
WNV- and USUV-positive polls and human cases of West Nile neuroinvasive disease (WNND). Mosquito pools tested positive for WNV, USUV, and WNND cases, with reference to the sampling period and region (Emilia-Romagna, red; Lombardia, green; Piemonte, yellow; Veneto, blue; and Friuli Venezia-Giulia, azure; black line represent the mosquito minimum infection rate).

More than 74% of the collected mosquitoes were tested for the presence of flavivirus (562,079 specimens sorted in 9,268 pools). WNV was detected in 180 (1.9%) pools, whereas USUV was found in 241 (2.6%) pools (Table 1). Minimum infection rates were evaluated on a fortnightly basis, obtaining a maximum value of 1.3 positive from 1,000 mosquitoes tested in the first half of August for WNV and of 2.1 in the second half of August for USUV (Fig 2). WNV-positive pools were sampled in Emilia-Romagna, Veneto, and Lombardia, whereas USUV was detected in these regions and in Piemonte (S1 Table). Both viruses were mainly detected in *Cx*. *pipiens* pools, and 28 pools tested positive for both viruses simultaneously. Other species found infected were *Ae*. *caspius* (2 USUV- and 1 WNV-positive pools) and *Cx*. *modestus* (1 USUV- and 1 WNV-positive pool). At the 185 stations sampled more than six times, both viruses were detected at 45 sites (24.3%), WNV only was detected at 19 sites (19.3%), and USUV only was detected at 48 sites (25.9%). Interestingly, a significant association was found between the detection of viruses at one site (χ^2^ = 15.343, p<0.001); a WNV-positive site has a 3.5 times greater probability to be positive for USUV compared to a negative site (Odds ratio = 3.5526, 95% CI = 1.857 to 6.797, z = 3.8296, p<0.001).

The first WNV-positive pools were sampled on July 3 at Modena and Venice provinces (Emilia-Romagna and Veneto regions, respectively) and on July 4 in Cremona Province (Lombardia region), whereas the last positive pool was sampled on October 1 in Rovigo Province (Veneto) (Fig 2). The first USUV-positive pool was sampled on June 25 in Treviso Province (Veneto region), whereas the last was sampled on October 9 in Verona Province (Veneto) (Fig 2). The peak of detection of positive pools was recorded in the first half of August for both viruses (Fig 2).

**Fig 3:**
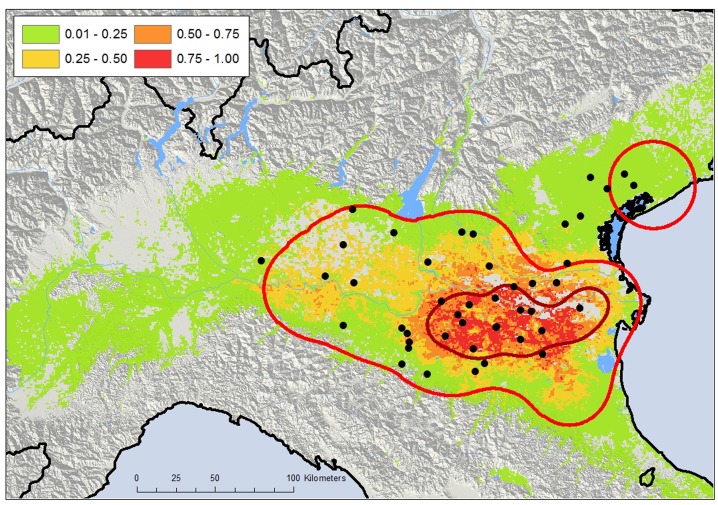
Nonlinear discriminant analysis (NLDA) and human cases of West Nile neuroinvasive disease (WNND). Localization of WNND cases (black dots) and NLDA analysis showing the modeled distribution probability of WNV circulation (see legend for probability) in northern Italy according to the results from 78 surveyed stations. The red lines represent the 95% and 50% volume contour of the WNV-circulation area estimated by kernel density estimation (KDE).

Two zones in which WNV circulated in 2013 were assessed by kernel density estimation (KDE) of the positive stations encompassing approximately 22,200 km^2^ (henceforth defined as the WNV-circulation area). The widest zone of approximately 20,100 km^2^ was located between Veneto, Lombardia, and Emilia-Romagna, whereas the smallest one (approximately 2,100 km^2^) was situated northeast of Veneto, in the same location as the WNV circulation area of the previous year [28] (Fig 1). The maximum intensity of the WNV circulation, highlighted by the 50% volume contour of KDE (50% VC), was recorded in an area of approximately 4,400 km^2^ between Emilia-Romagna, Veneto, and Lombardia (Fig 1). The first human WNND case (detected in the Veneto region) reported the onset of symptoms on July 21 and was followed by 44 other human cases (20 in Emilia-Romagna, 15 in Veneto, and 10 in Lombardia) (Fig 2). Of the WNND human cases, 39/45 lived within the estimated WNV-circulation area (Fig 3).

The KDE of the USUV-positive stations produced an area of 30,600 km^2^, which overlapped 19,200 km^2^ (87%) of the circulation area for the WNV (Fig 1). The more intense circulation of the USUV was recorded in 4 separated zones; the wider of these zones, which was 5,400 km^2^, overlapped 3,200 km^2^ of the WNV 50% VC (Fig 1).

The altitude of the 183 sites in the circulation area for WNV ranged from -4 to 311 m a.s.l., and 22 sites were in hilly areas. Although these areas showed less favorable ecological conditions for the WNV circulation compared to the plain, 1 of these sites (128 m a.s.l.) was WNV-positive (4.5%). The 230 sites in the USUV-KDE had an altitude between -4 and 444 m a.s.l.; of these sites, 45 were in hilly areas, and 8 (from 53 to 214 m a.s.l.) were USUV-positive (17.8%).

The monthly average of *Cx*. *pipiens* mosquitoes collected per site from June to September was significantly higher in sites inside the circulation area for WNV than in sites outside that area (Table 2). Inside the circulation area, a statistically significant difference was noted in the average number of specimens collected monthly between the WNV-positive and WNV-negative sites but only in September (an average of 126 specimens, SD 167, in 49 negative sites vs. 190 specimens, SD 384, in 61 positive sites; Kruskal-Wallis χ^2^ = 6.32, p<0.05).

**Table 2:** Monthly average of *Culex pipiens* specimens, temperatures, cumulative precipitation, evapotranspiration (Evapo.), and enhanced vegetation index (EVI) value inside and outside the WNV-circulation area estimated by KDE, *p<0.05 **p<0.01 according to a Kruskal-Wallis test. The standard deviations and number of observations are reported in S2 Table.

	*Cx*. *pipiens* specimens		Temperature°C		Precipitation mm		Evapo. mm		EVI	
	In	Out	In	Out	In	Out	In	Out	In	Out
April	-	-	22.1	22.1	92	108*	59	52**	0.34	0.29**
May	177	85*	24.0	22.9**	123	187*	86	78**	0.36	0.33**
June	955	296*	32.1	30.2**	32	38	120	110**	0.34	0.35**
July	848	268*	33.2	31.8**	24	40*	130	111**	0.39	0.40**
August	359	148*	31.2	29.8**	63	74*	89	83**	0.39	0.42**
September	161	35*	28.0	25.7**	32	67*	59	53**	0.32	0.38**
October	9	5	18.9	18.1**	115	96*	25	22**	0.26	0.32**

In the period of April through September the circulation area for WNV was characterized, on a monthly basis, by high temperatures and evapotranspiration values and the lowest precipitation (Table 2, S2 Table). The monthly enhanced vegetation index (EVI), a satellite index linked to the photosynthetic activity and vegetation composition, also differed between the two zones, with the lowest value inside the WNV-circulation area in the period for June through October (Table 2, S2 Table) and the highest value inside the area in the two previous recorded months.

The nonlinear discriminant analysis (NLDA) model largely confirmed the circulation area estimated by the KDE, particularly the more intense WNV-circulation area (Fig 3). Variables that contribute more significantly to the model include thermal and vegetation indexes (S3 Table). The accuracy of the NLDA circulation model was “excellent,” as judged by Cohen’s kappa (0.8285+/-0.0584); both the sensitivity (correct presence percentage) and specificity of the model (correct absence percentage) exceeded 0.90 (S3 Table).

Among the WNV detections in the positive pools, 170 belonged to lineage II, highlighting the intense circulation of a lineage of WNV not involved in the previous outbreaks in the surveyed areas. The 76 longest and cleanest WNV lineage II sequences (32 from Emilia-Romagna and 44 from Veneto) were aligned to obtain alignments of 200 nucleotides that were part of the NS5 gene of flaviviruses (S1 File). Of these sequences, 73 showed a complete identity, whereas a single point mutation was observed in 3 sequences. Among the sequences available in the GenBank (GB) database, the consensus sequence obtained by this alignment showed the highest identity with sequences from human cases recorded in the Pianura Padana in 2013 and with sequences from mosquitoes detected in 2011–12 in Veneto and Friuli Venezia-Giulia; identities of 99% were obtained for sequences detected between 2004 and 2011 in Hungary, Austria, Serbia, and Greece (S4 Table). Interestingly, the WNV lineage I sequences were also detected in two pools collected in Rovigo Province, an area in which this lineage had circulated in previous years; these sequences showed a maximum identity with sequences already detected in the surveyed area (S4 Table). An alignment with sequences homologous with WNV sequences was also obtained between 84 USUV sequences (57 from Veneto and Friuli Venezia-Giulia, 25 from Emilia-Romagna, one from Lombardia, and one from Piemonte); the identity between these sequences ranged from 98% to 100%. The obtained consensus had a complete identity with sequences already detected in Pianura Padana in 2009 and 2010, as well as an identity of 99.5% with the sequence from a human case detected in the surveyed area in 2009 (S5 Table).

## Discussion

The entomological surveillance system detected a wide circulation of WNV, characterizing the dynamics and extension of the 2013 outbreak in Pianura Padana. Virus detection in mosquitoes, achieved at the beginning of July, clearly precedes the appearance of human cases, which were recorded from the end of July, as reported elsewhere [20, 21, 22]. The entomological monitoring highlighted an intense viral circulation in a particular area, confirmed also by the high number of WNND cases in this area, demonstrating the ability of the surveillance system to locate the extent of viral circulation (Fig 3). The results obtained here showed a good performance of the entomological surveillance program for assessing the geographical spread of the viruses in a wide area. These results also demonstrated that if the sampling effort is adequate, this system is able to detect WNV well before the onset of human cases, allowing the organization of appropriate preventive public health strategies.

The majority of the WNV sequences detected belonged to lineage II, a strain not previously linked with human disease in the area. Lineage I sequences, which circulated in 2008–2012, were still detected in low amounts in the 2013 surveillance, indicating the likely establishment of these viruses and the risk of a future reemergence. The lineage II virus detected was closely related to those circulating in Europe, from Hungary to Greece. The hypothesis of a progressive expansion of this WNV strain from Eastern European countries to neighboring countries, as far as Italy, can explain this situation.

The surveillance system confirms *Cx*. *pipiens* as the main vector of the virus in northern Italy. The occasional detection of WNV in *Cx*. *modestus* and *Ae*. *caspius* does not represent proof of the involvement of the two species in the virus cycle. However, *Cx*. *modestus* has been demonstrated to be an efficient laboratory WNV vector [29]; conversely, an active role of *Ae*. *caspius* seems more unlikely, due to its poor vector competence for WNV [29] and feeding preference for mammals rather than birds [30, 31]. Moreover, at both sites in which these pools were sampled, *Cx*. *pipiens*-positive pools were also collected, confirming the primary role of this mosquito in the circulation of WNV in the surveyed area.

The abundance of *Cx*. *pipiens* (estimated as a monthly average of sampled mosquitoes per station) was highest inside the WNV-circulation area (compared to the remaining part of Pianura Padana), but in that area, a close relationship between the mosquito abundance and the viral circulation was not observed. These data suggest that the number of mosquitoes must exceed a certain threshold to trigger a virus circulation intense enough to provoke human cases; according to the monthly average of collected specimens outside the WNV-circulation area in June and July, the value of 300 *Cx*. *pipiens* per trap per night was empirically estimated to correspond to the minimal risk threshold for human cases in the described surveillance system. The surveillance system was also able to detect the non-target USUV. This virus is closely related to WNV, and their differentiation is needed in a surveillance plan to avoid false positive results [32]. The affinity between these two viruses is also confirmed by the shared main vector, *Cx*. *pipiens*, and the similarity in ecological needs, which is demonstrated by the positive relationship between the detection of the two viruses at a particular site. The circulation areas of the two viruses largely overlapped, confirming their co-circulation in Pianura Padana, as previously reported [23]; moreover, although the majority of the monitored sites were in the plain area, both viruses were detected in hilly areas as well (although with a different prevalence in the tested mosquitoes). Despite these similarities, the two viruses showed different temporal patterns of circulation in the surveyed area: WNV had been circulating discontinuously, with different strains and in different locations, over the years [11], whereas USUV has been persistently detected at similar levels since 2009 [33], showing a continuous circulation. Moreover, the USUV field-detected sequences showed more heterogeneity than the WNV sequences, probably due to the longer circulation of USUV in the surveyed area or to the presence of different strains. These differences raise questions about the epidemiologic characteristics responsible for producing the different patterns of spread and abundance of the two viruses.

The WNV lineage II strain, primarily detected in the 2013 outbreak, was already present in the surveyed area in 2011 and 2012 [12, 21], suggesting the silent circulation of the virus in the sylvatic cycle for several seasons before the appearance of the disease. In this scenario, the characterization of those factors able to provoke the spillover of the virus to affect humans and horses is crucial. The system in place allowed evaluation of the influence of various biotic and abiotic factors on WNV circulation. The WNV circulation area was characterized by minor precipitation and the highest evapotranspiration and temperatures between May and September, compared to the remaining part of Pianura Padana. The EVI indexes indicate a higher photosynthetic activity in April through May and then a lower photosynthetic activity in June through October inside the WNV-circulation area, with respect to the remainder of the plain. These results indicate that the circulation of WNV was favored by less rainy and warmer conditions, which led to the hypothesis of a trigger capacity of these conditions on the outbreak onsets.

The link between WNF and high temperature had already been observed in Europe on a continental scale [34–36]; moreover, the amount of water in the ecosystem was negatively correlated with WNF incidence [36]. Due to these characteristics, climate change is likely to exacerbate the recrudescence of this virus in Europe [37]. The data obtained in this study are concordant with these previous results and findings and seem to indicate that in an ecologically homogeneous territory, high temperature and drier conditions (characterized by low rainfall and high evapotranspiration) are able to increase WNV circulation and then cause viral spillover to infect dead-end hosts.

## Materials and Methods

### Surveyed area

Extending approximately 46,000 km^2^, the Pianura Padana (or Pianura Padano-Veneta) is the widest plain of Italy, which includes the Po River Basin as well as the Veneto and Friuli plains, which were created by other important rivers. The Pianura Padana can be considered a homogeneous territory with characteristic morphology and hydrology, bounded by the Alps to the north and the Apennines to the south. This plain declines in altitude slowly from west to east to the Adriatic Sea, where it has a breadth of approximately 270 km. Pianura Padana is densely populated, with 500 inhabitants per km^2^ and a total population of approximately 20 million; the plain is administratively subdivided into five regions (Emilia-Romagna, Friuli Venezia-Giulia, Lombardia, Piemonte, and Veneto). Productive and urban areas are abundant, and the environment is strongly influenced by human activity, characterized by intensive agriculture, with a few hedges, scattered uncultivated areas (natural protected areas, riverbeds, and disused quarries), and a complex irrigation network. Vineyards, orchards, and poplar cultivations are locally abundant, as are rice fields. The climate of this area is described as warm temperate and fully humid, with hot/warm summers, according to the Köppen-Geiger climate classification [38].

### Mosquito sampling

Mosquito-based surveillance systems were based on the collection of insects at 286 stations from May to October with attractive traps: carbon dioxide-baited traps were active in all regions at 262 sites [39], 16 gravid traps were used in Emilia-Romagna (John W. Hock Company), and 8 BG sentinel traps (Biogents), carbon dioxide baited, were used in Piemonte. Each station was geo-referenced, and traps worked for one night, from 17:00 to 9:00 the next day; only the BG sentinel worked for a 24-hour period. Sampled mosquitoes were identified at the species level with morphological keys [30, 31, 40] and then pooled according to station and date of sampling, with a maximum of 200 specimens per pool. The traditional classification of mosquitoes was adopted in this work, as proposed by Savage and Strickman [41]. In Emilia-Romagna and Lombardia, the sampling frequency was fortnightly, with a maximum of 5 pools tested for species/sampling (for a maximum of 1,000 specimens). Over the regional system, a supplementary program operated at 24 sites in Emilia-Romagna (Modena Province); moreover, one-night samplings were conducted in a hilly part of the region. In Lombardia, surveillance started at the beginning of September, but four sites were monitored for other purposes from the beginning of July. In Veneto and Friuli Venezia-Giulia, sampling began in May and was conducted weekly; mosquitoes were analyzed for flaviviruses beginning with samples from the last week of June, with a maximum of 50 specimens per pool. In Piemonte, surveillance began fortnightly at the beginning of July, with all specimens being tested, and then, beginning in August, a maximum of 2 pools (50 specimens each) were tested from each species/sampling. Monthly averages of sampled *Cx*. *pipiens* mosquitoes were calculated for sites with two or more samples in the month of reference.

### Virological analysis

The methods of extraction, reverse transcription, and PCR protocols are extensively described elsewhere [12, 17, 19]. Briefly, RNA was extracted from pools of mosquitoes using Trizol reagent (or equivalent) or with a commercial kit (NucleoSpin 96 RNA kit; Machery-Nagel, Duren, Germany) and reverse transcribed (RT) using random primers. After RT reactions, the samples were subjected to two real-time PCR protocols for the detection of WNV and USUV and a screening PCR for the presence of flaviviruses, which targeted 250 nucleotides of the conserved region of the NS5 gene [42], and the obtained amplicons were sequenced for virus identification. The obtained sequences were aligned by the ClustalW algorithm implemented in MEGA 6 software [43], and the alignment was refined manually. Consensus sequences of the aligned sequences were obtained manually, with nucleotides more abundant than 80% being selected for every position. The number of base differences between sequences was obtained by the pairwise distance computation of Mega 6 software [43].

### Spatial analysis

Two spatial analysis approaches were used to identify areas with high WNV circulation: one exploratory analysis based on KDE (kernel density estimation) and one statistical approach based on NLDA (nonlinear discriminant analysis). (I) KDE analysis is a geospatial technique based on the kernel function (Gaussian function in this case) used to create a surface to indicate the magnitude of the investigated events. Each of the 78 positive stations (stations with at least a positive pool in the season) was considered as a single event (data from the Emilia-Romagna supplementary program were not considered for this analysis). The optimum bandwidth size of 20 km was calculated using Biased Cross Validation (BCV) for WNV data. The area of WNV circulation was estimated by the 95% volume contour of the WNV KDE, representing the area that would contain 95% of the points used to generate the KDE (Fig 3). Similarly, the KDE area was also estimated for USUV, considering 101 positive stations and a bandwidth size of 10 km, calculated by BCV (Fig 3). (II) NLDA [44] is a spatial analysis technique that produces a geospatial model of a phenomenon. NLDA works with categorical data. In this case, the predicted variable was the presence/absence of WNV-positive pools, and 122 climatic and environmental variables, available on-line [45, 46, 47], were screened to individuate the best predictors; these variables are described in S3 Table. NLDA was applied to 204 surveyed sites, with more than 3 samplings during the season (78 positive sites and 126 negative sites). The bootstrapping of 100 models was applied, and models were averaged to describe the WNV circulation probability. The set of the more predictive variables were then obtained in each model in a stepwise inclusion manner to minimize the Akaike Information Criterion (AICc) corrected for small samples [48]. Variables were then ranked to obtain the set of 10 variables that together better explicate the data, not simply the 10 best-predicting variables [49]. NLDA was performed using Vecmap™ (www.vecmap.com).

The limit of the plain area was manually defined according to a digital elevation model and satellite images. Four meteorological parameters, recorded in 2013, were obtained for the defined plain area: the monthly average of the daytime land surface temperature and the enhanced vegetation index (EVI) at 0.05 degree, 1 km resolution (satellite data obtained by MODIS Images available at http://ladsweb.nascom.nasa.gov/data/search.html); the cumulated evapotranspiration data at a 3-km resolution (obtained by addition of daily EUMETSAT satellite data available at https://landsaf.meteo.pt); and the monthly cumulated precipitation data from 187 selected meteorological stations obtained online in sites of the five Regional Environmental Agencies (ARPAs) located on the surveyed territory. Municipalities of the domiciles of the WNND human patients were obtained from the Health Regional Services, and centroids of these municipalities were used as a reference location with respect to the WNV-circulation area. Geographical data extractions and elaborations, KDE and maps production were performed using ESRI ArcGIS 9.3 and the Spatial Analyst extension.

### Statistical analysis

Minimum infectious rates were calculated fortnightly from June to September, assuming one positive mosquito per positive pool over the number of tested mosquitoes. Differences in the average of weather data and in the monthly average of the *Cx*. *pipiens* specimens per site, inside and outside the area of the 95% KDE of WNV, were compared via a nonparametric Kruskal-Wallis test for equality of populations. Additionally, the monthly averages of *Cx*. *pipiens* specimens per site, between positive and negative sites inside the area of 95% KDE of WNV, were compared via a Kruskal-Wallis test. The presence/absence data of WNV and USUV per site were compared using a χ^2^ test, and the odds ratio was evaluated. Statistical elaboration was performed using Intercooled Stata 7.0 software (Stata Corporation, College Station, TX, USA). The significance level was set at p<0.05.

### Ethics statement

Mosquitoes were sampled from public land according to the guidelines of the WNV National Surveillance plan and the official notes of the Regional Health Authorities (RHAs) who provided implicit authorization to perform the sampling on public land. Mosquitoes sampled from private land were collected after verbal informed consent was received from the landowners.

Data on human cases were acquired by RHAs from ordinary diagnostic activity and were treated and analyzed anonymously, based on official notes from RHAs and National HA.

## Supporting Information

S1 Fig Map of sampled sites. (PNG)

S2 Fig Average monthly collection of *Culex pipiens* in surveyed stations with reference to the month of collection. (PDF)

S1 File FASTA file with alignment of the 78 WNV and 84 USUV field sequences (200 bp of the NS5 gene). (TXT)

S1 Table Sampled and tested mosquitoes with reference to the sampling region. (DOCX)

S2 Table Weather condition and *Culex pipiens* specimens inside and outside the WNV circulation area. (DOCX)

S3 Table Average statistics of the NLDA model and list of screened variables. (DOCX)

S4 Table Identity between the consensus of the WNV field-detected sequences and other homologous sequences. (DOCX)

S5 Table Identity between the consensus of the USUV field-detected sequences and other homologous sequences. (DOCX)
