# Correction: West Nile Virus Surveillance in 2013 via Mosquito Screening in Northern Italy and the Influence of Weather on Virus Circulation

**DOI:** 10.1371/journal.pone.0146436

**Published:** 2015-12-30

**Authors:** The PLOS ONE Staff

There are formatting errors in Table 2; the publisher apologizes for the errors. Please see the corrected Table 2 here.

**Table 2:** Monthly average of *Culex pipiens* specimens, temperatures, cumulative precipitation, evapotranspiration (Evapo.), and enhanced vegetation index (EVI) value inside and outside the WNV-circulation area estimated by KDE, *p<0.05 **p<0.01 according to a Kruskal-Wallis test. The standard deviations and number of observations are reported in S2 Table.

	*Cx*. *pipiens* specimens		Temperature°C		Precipitation mm		Evapo. mm		EVI	
	In	Out	In	Out	In	Out	In	Out	In	Out
April	-	-	22.1	22.1	92	108*	59	52**	0.34	0.29**
May	177	85*	24.0	22.9**	123	187*	86	78**	0.36	0.33**
June	955	296*	32.1	30.2**	32	38	120	110**	0.34	0.35**
July	848	268*	33.2	31.8**	24	40*	130	111**	0.39	0.40**
August	359	148*	31.2	29.8**	63	74*	89	83**	0.39	0.42**
September	161	35*	28.0	25.7**	32	67*	59	53**	0.32	0.38**
October	9	5	18.9	18.1**	115	96*	25	22**	0.26	0.32**
