# Supplementary material for: West Nile Virus Surveillance in 2013 via Mosquito Screening in Northern Italy and the Influence of Weather on Virus Circulation
Source: PLoS One. 2015 Oct 21;10(10):e0140915. doi: 10.1371/journal.pone.0140915 (PMC4619062; doi:10.1371/journal.pone.0140915)

S2 Figure. Average of monthly collection of *Culex pipiens* in surveyed stations referred to month of collection.

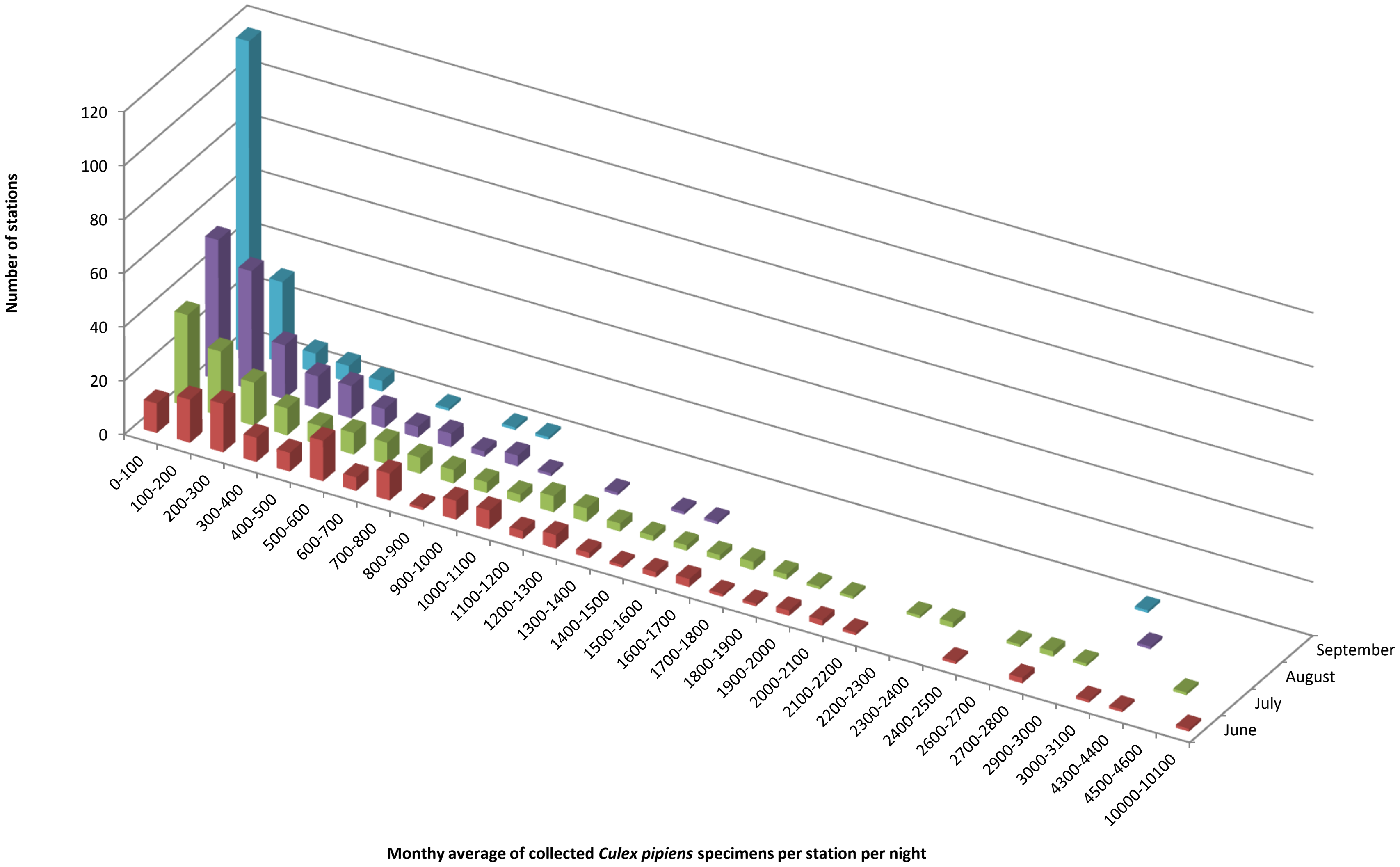

Supplement: S2 Fig — (PDF) [file pone.0140915.s002.pdf]
