# Supplementary material for: West Nile Virus Surveillance in 2013 via Mosquito Screening in Northern Italy and the Influence of Weather on Virus Circulation
Source: PLoS One. 2015 Oct 21;10(10):e0140915. doi: 10.1371/journal.pone.0140915 (PMC4619062; doi:10.1371/journal.pone.0140915)
Supplement: S1 Table — (DOCX) [file pone.0140915.s004.docx]

**S1 Table. . Sampled and tested mosquitoes with reference to sampling region.** Number of collected (N) & tested mosquitoes (Nt), number of pools (Np), WNV/USUV (W/U) positive pools for mosquito species and for surveyed regions.

|  | *Emilia-Romagna* | | *Veneto* | | *Friuli*  *Venezia-Giulia* | | *Piemonte* | | *Lombardia* | | *Total* | | |
| --- | --- | --- | --- | --- | --- | --- | --- | --- | --- | --- | --- | --- | --- |
| Species | N/Nt | Np (W/U) | N/Nt | Np (W/U) | N/Nt | Np (W/U) | N/Nt | Np (W/U) | N/Nt | Np (W/U) | N | Nt | Np (W+/U+) |
| *Ae. cinereus/geminus* | 70/70 | 1 | 28/12 | 2 |  |  | 1/1 | 1 | 12/12 | 3 | 111 | 95 | 6 |
| *Ae. (Och.) cantans* |  |  | 118/ 69 | 26 |  |  | 53/53 | 5 |  |  | 171 | 74 | 26 |
| *Ae.(Och.) caspius* | 20,072/ 18,802 | 303 (1/2) | 13,677/ 9,752 | 466 | 465/ 358 | 33 | 7,150/ 5,279 | 186 | 2,744/ 2,744 | 42 | 44,108 | 31,842 | 844 (1/2) |
| *Ae.(Och.) detritus* |  |  | 692/ 116 | 18 | 1/1 | 1 |  |  |  |  | 693 | 117 | 19 |
| *Ae.(St.) albopictus* | 1,993/ 1,567 | 139 | 1,039/ 865 | 232 | 504/3 | 55 | 1,002/ 1,002 | 92 | 121/121 | 37 | 4,659 | 2,648 | 463 |
| *Ae. geniculatus* | 88/72 | 2 | 33/ 9 | 3 | 64/36 | 2 | 26/26 | 10 | 3/3 | 2 | 214 | 130 | 9 |
| *Ae. (Hu) koreicus* |  |  | 26/6 | 5 |  |  |  |  |  |  | 26 | 6 | 5 |
| *Ae. vexans* | 9,938/ 8,857 | 119 | 1,034/ 332 | 83 | 170/93 | 17 | 926/ 530 | 44 | 2,368/ 2,368 | 38 | 14,436 | 11,694 | 257 |
| *An. claviger./petragnani* |  |  | 10/5 | 5 | 6/5 | 3 |  |  |  |  | 16 | 10 | 8 |
| *An. maculipennis s.l.* | 522/ 25 | 2 | 822/637 | 118 | 20/19 | 11 | 2,129/ 1,539 | 71 | 1,952/ 1,952 | 25 | 5,445 | 2,704 | 156 |
| *An. plumbeus* | 7/0 |  | 17/14 | 9 | 28/18 | 3 | 3/3 | 3 |  |  | 55 | 35 | 12 |
| *Cq. richiardii* | 63/54 | 5 | 266/187 | 30 | 6/6 | 4 |  |  |  |  | 335 | 247 | 39 |
| *Cs. annulata* | 34/0 |  | 186/182 | 64 | 17/12 | 10 |  |  | 9/9 | 8 | 246 | 203 | 82 |
| *Cx. modestus* | 1,360/ 1,295 | 40(1/1) | 101/79 | 25 |  |  | 718/ 371 | 24 | 52/52 | 10 | 2,231 | 1,450 | 75 (1/1) |
| *Cx. pipiens* | 395,034/ 279,922 | 1995 (115/114) | 257,215/ 208,545 | 4852 (57/116) | 10,409/ 8,538 | 284 (0/6) | 8,475/ 6,845 | 241 (0/1) | 13,527/ 13,527 | 125 (4/1) | 684,660 | 510,773 | 7256 (179/238) |
| *Cx. territans* |  |  | 42/ 39 | 5 |  |  |  |  |  |  | 42 | 39 | 5 |
| Other species | 1/0 |  | 5/5 | 3 |  |  | 1/1 | 1 | 7/7 | 3 | 14 | 13 | 13 |
| Total | 429,1827/ 310,664 | 2606/ 117/117 | 275,308/ 220,851 | 5945 | 11,693/ 9,092 | 424 | 20,484/ 15,650 | 678 | 20,795/ 20,795 | 293 | 757,461 | 562,079 | 9,268/ 181/241 |

*Ae. (Och.)berlandi (1), Ae.(Och.)sticticus (3,) Ae.(Och.) flavescens (1), Aedes spp.(7), Cs. subchorea (1), Cx. hortensis (1).*
