# Supplementary material for: West Nile Virus Surveillance in 2013 via Mosquito Screening in Northern Italy and the Influence of Weather on Virus Circulation
Source: PLoS One. 2015 Oct 21;10(10):e0140915. doi: 10.1371/journal.pone.0140915 (PMC4619062; doi:10.1371/journal.pone.0140915)
Supplement: S2 Table — (DOCX) [file pone.0140915.s005.docx]

**S2 Table. Weather condition and *Cx. pipiens* specimens inside and outside the WNV circulation area.** Monthly average of *Culex pipiens* specimens, temperatures, cumulated precipitation, evapotranspiration EVI value inside and outside the WNV circulation area estimated by KDE, with reference to standard deviation (SD) and number of observations, *p<0.05 **p<0.01.

|  | Average of *Cx. pipiens* specimens | | | | Temperature | | | | Precipitation | | | | Evapotranspiration | | | | EVI | | | | | |
| --- | --- | --- | --- | --- | --- | --- | --- | --- | --- | --- | --- | --- | --- | --- | --- | --- | --- | --- | --- | --- | --- | --- |
|  | In | | Out | | In | | Out | | In | | Out | | In | | Out | | In | | | Out | | |
|  | Sites | N(SD) | Sites | N(SD) | Points | °C(SD) | Points | °C(SD) | s | mm(SD) | Sites | mm(SD) | Points | mm(SD) | Points | mm(SD) | Points | Value(SD) | Points | | | Value(SD) |
| April |  | - |  | - | 922 | 22,1 (1,4) | 1121 | 22,1 (1,5) | 83 | 92 (36) | 101 | 108 (48)* | 1228 | 59 (9) | 1437 | 52 (11)** | 922 | 0,34 (0,07) | 1121 | | 0,29 (0,09)** | |
| May | 23 | 177 (161) | 20 | 85 (98)* | 922 | 24,0 (1,1) | 1121 | 22,9 (1,5)** | 85 | 123 (67) | 101 | 187 (86)* | 1228 | 86 (12) | 1437 | 78 (16)** | 922 | 0,36 (0,07) | 1121 | | 0,33 (0,09)** | |
| June | 104 | 955 (1156) | 28 | 296 (322)* | 922 | 32,1 (1,6) | 1121 | 30,2 (2,0)** | 85 | 32 (17) | 102 | 38 (29) | 1228 | 120 (18) | 1437 | 110 (26)** | 922 | 0,34 (0,06) | 1121 | | 0,35 (0,07)** | |
| July | 106 | 848 (787) | 46 | 268 (359)* | 922 | 33,2 (1,5) | 1121 | 31,8 (2,2)** | 85 | 24 (21) | 102 | 40 (29)* | 1228 | 130 (24) | 1437 | 111 (32)** | 922 | 0,39 (0,05) | 1121 | | 0,40 (0,08)** | |
| August | 110 | 359 (380) | 48 | 148 (250)* | 922 | 31,2 (2,1) | 1121 | 29,8 (2,4)** | 85 | 63 (27) | 102 | 74 (33)* | 1228 | 89 (22) | 1437 | 83 (27)** | 922 | 0,39 (0,08) | 1121 | | 0,42 (0,10)** | |
| September | 110 | 161 (307) | 48 | 35 (37)* | 922 | 28,0 (2,2) | 1121 | 25,7 (1,8)** | 85 | 32 (18) | 102 | 67 (78)* | 1228 | 59 (11) | 1437 | 53 (14)** | 922 | 0,32 (0,08) | 1121 | | 0,38 (0,08)** | |
| October | 30 | 9 (11) | 23 | 5 (8) | 922 | 18,9 (0,9) | 1121 | 18,1 (1,2)** | 83 | 115 (30) | 101 | 96 (27)* | 1228 | 25 (4) | 1437 | 22 (5)** | 922 | 0,26 (0,07) | 1121 | | 0,32 (0,07)** | |

**S3 Table. Average statistic of the NLDA model.**

Top 10 variables average rank

Rank Variable

7,400 WC1KTMEAN8: WC1KTMEAN8

7,600 ED1803A1: Middle infra-red amplitude 1

8,000 WC1KTMEAN2: WC1KTMEAN2

8,000 WC1KTMEAN6: WC1KTMEAN6

8,100 ED1803VR: Middle infra-red variance

8,500 ED1815A3: EVI amplitude 3

8,600 ED1807A3: Daytime LST amplitude 3

8,700 ED1807MN: Daytime LST minimum

8,800 ED1814A2: NDVI amplitude 2

9,000 ED1814VR: NDVI variance

Average nlda accuracy statistics:

Kappa: 0.8285 +/- 0.0584

AUC: 0.9693 +/- 0.0317

Sensitivity: 0.9015 +/- 0.0402

Specificity: 0.9569 +/- 0.0306

**S4 Table. Identity between the WNV field detected sequences and other homologous sequences.** Number of base differences (lower left) and percentage of identity (upper right) between the consensus of WNV field detected sequences and other homologous sequences deposed in Gen Bank (GB). Alignment of 200 base pairs of the NS5 gene, * sequences detected in the surveyed area.

|  | Strain | Lin | Country | Year | Origin | GB | 1 | 2 | 3 | 4 | 5 | 6 | 7 | 8 | 9 | 10 | 11 | 12 |
| --- | --- | --- | --- | --- | --- | --- | --- | --- | --- | --- | --- | --- | --- | --- | --- | --- | --- | --- |
| 1 | This study | II | Italy * | 2013 | Mosquito | - |  | 99.5 | 99.0 | 99.0 | 99.0 | 99.0 | 99.0 | 99.0 | 81.0 | 80.5 | 80.0 | 80.0 |
| 2 | 2436/12_WNlin2 | II | Italy * | 2012 | Mosquito | - | 1 |  | 98.5 | 98.5 | 98.5 | 98.5 | 98.5 | 98.5 | 81.0 | 80.5 | 80.0 | 80.0 |
| 3 | Rovigo/34.1 | II | Italy * | 2013 | Human | KF647248 | 2 | 3 |  | 100 | 100 | 100 | 100 | 100 | 80.0 | 79.5 | 79.0 | 79.0 |
| 4 | Hungary/04 | II | Hungary | 2004 | Bird | DQ116961 | 2 | 3 | 0 |  |  | 100 | 100 | 100 | 80.0 | 79.5 | 79.0 | 79.0 |
| 5 | Novi_Sad-2010 | II | Serbia | 2010 | Mosquito | KC496016 | 2 | 3 | 0 | 0 |  | 100 | 100 | 100 | 80.0 | 79.5 | 79.0 | 79.0 |
| 6 | Austria/2008 | II | Austria | 2008 | Bird | KF179640 | 2 | 3 | 0 | 0 | 0 |  | 100 | 100 | 80.0 | 79.5 | 79.0 | 79.0 |
| 7 | Italy/2011/AN-2 | II | Italy | 2011 | Human | JN858070 | 2 | 3 | 0 | 0 | 0 | 0 |  | 100 | 80.0 | 79.5 | 79.0 | 79.0 |
| 8 | Nea_Santa- 2010 | II | Greece | 2010 | Human | HQ537483 | 2 | 3 | 0 | 0 | 0 | 0 | 0 |  | 80.0 | 79.5 | 79.0 | 79.0 |
| 9 | This study | I | Italy * | 2013 | Mosquito | - | 38 | 38 | 40 | 40 | 40 | 40 | 40 | 40 |  | 99.0 | 98.0 | 98.5 |
| 10 | Italy/2008/M-203204 | I | Italy * | 2008 | Bird | JF719066 | 39 | 39 | 41 | 41 | 41 | 41 | 41 | 41 | 2 |  | 99.0 | 99.5 |
| 11 | Italy/2011/Livenza | I | Italy * | 2011 | Human | JQ928174 | 40 | 40 | 42 | 42 | 42 | 42 | 42 | 42 | 4 | 2 |  | 98.5 |
| 12 | Italy/2011/Piave | I | Italy * | 2011 | Human | JQ928175 | 40 | 40 | 42 | 42 | 42 | 42 | 42 | 42 | 3 | 1 | 3 |  |

**S5 Table. Identity between the USUV field detected sequences and other homologous sequences.** Number of base differences (lower left) and percentage of identity (upper right) between the consensus of USUV field detected sequences and other homologous sequences deposed in Gen Bank (GB). Alignment of 200 base pairs of the NS5 gene, * sequences detected in the surveyed area.

|  | Strain | Country | Year | Origin | GB | 1 | 2 | 3 | 4 | 5 | 6 | 7 | 8 | 9 | 10 | 11 | 12 |
| --- | --- | --- | --- | --- | --- | --- | --- | --- | --- | --- | --- | --- | --- | --- | --- | --- | --- |
| 1 | This study | Italy | 2013 | Mosquito | - |  | 100 | 99.5 | 99.0 | 99.0 | 98.5 | 98.0 | 97.5 | 97.5 | 97.5 | 97.0 | 96.5 |
| 2 | USU-RE-m7/2010 | Italy* | 2010 | Mosquito | JF834591 | 0 |  | 99.5 | 99.5 | 99.5 | 99.0 | 98.5 | 98.0 | 98.0 | 98.0 | 97.0 | 96.5 |
| 3 | Bologna 2009 | Italy* | 2009 | Human | HM569263 | 1 | 1 |  | 99.0 | 99.0 | 98.5 | 98.0 | 97.5 | 97.5 | 97.5 | 97.0 | 96.5 |
| 4 | Vienna_2001 | Vienna | 2001 | Bird | AY453411 | 2 | 1 | 2 |  | 100 | 98.5 | 99.0 | 98.5 | 97.5 | 98.5 | 96.5 | 96.0 |
| 5 | Meise H | Austria | 2002 | Bird | JQ219843 | 2 | 1 | 2 | 0 |  | 98.5 | 99.0 | 98.5 | 97.5 | 98.5 | 96.5 | 96.0 |
| 6 | m2080-NO/ITA/2011 | Italy* | 2011 | Mosquito | KF882514 | 3 | 2 | 3 | 3 | 3 |  | 98.5 | 98.0 | 98.0 | 98.0 | 97.0 | 96.5 |
| 7 | Milan 2006 | Italy* | 2006 | Bird | JX473242 | 4 | 3 | 4 | 2 | 2 | 3 |  | 98.5 | 97.5 | 98.5 | 96.5 | 96.0 |
| 8 | BAT2USUTU-BNI | Germany | 2013 | Bat | KJ859683 | 5 | 4 | 5 | 3 | 3 | 4 | 3 |  | 97.0 | 100 | 96.0 | 96.5 |
| 9 | ArD192495 | Senegal | 2007 | Mosquito | KC754957 | 5 | 4 | 5 | 5 | 5 | 4 | 5 | 6 |  | 97.0 | 99.0 | 97.5 |
| 10 | BH65/11-02-03 | Germany | 2011 | Bird | HE599647 | 5 | 4 | 5 | 3 | 3 | 4 | 3 | 0 | 6 |  | 96.0 | 96.5 |
| 11 | HB81P08 | C. African Rep. | 1981 | Human | KC754955 | 6 | 6 | 6 | 7 | 7 | 6 | 7 | 8 | 2 | 8 |  | 97.5 |
| 12 | SAAR-1776 | South Africa | 1959 | Mosquito | AY453412 | 7 | 7 | 7 | 8 | 8 | 7 | 8 | 7 | 5 | 7 | 5 |  |
