# Supplementary material for: West Nile Virus Surveillance in 2013 via Mosquito Screening in Northern Italy and the Influence of Weather on Virus Circulation
Source: PLoS One. 2015 Oct 21;10(10):e0140915. doi: 10.1371/journal.pone.0140915 (PMC4619062; doi:10.1371/journal.pone.0140915)
Supplement: S3 Table — (DOCX) [file pone.0140915.s006.docx]

**S3 Table. NLDA model.**

NLDA [44] is a spatial analysis technique that produce a geospatial model of a phenomenon.

Presence/absence of WNV positive pools in a site was the predicted variables, other 122 predictor variables were organized as raster type files and for each trap location the pixel values of the environmental variables were extracted.

These data were obtained from:

1) Eden Next Data Archive (<http://www.edenextdata.com>) (ED), files free available as MODIS Fourier Processed Imagery 2001-08 which include [45]:

Middle infra-red mean

Middle infra-red amplitude of annual cycle

Middle infra-red amplitude of bi-annual cycle

Middle infra-red amplitude of tri-annual cycle

Middle infra-red variance in annual cycle

Middle infra-red variance in bi-annual cycle

Middle infra-red variance in tri-annual cycle

Middle infra-red combined variance in annual, bi-annual, and tri-annual cycles

Middle infra-red minimum

Middle infra-red maximum

Middle infra-red phase of annual cycle

Middle infra-red phase of bi-annual cycle

Middle infra-red phase of tri-annual cycle

Middle infra-red variance in raw data

Daytime land surface temperature mean

Daytime land surface temperature amplitude of annual cycle

Daytime land surface temperature amplitude of bi-annual cycle

Daytime land surface temperature amplitude of tri-annual cycle

Daytime land surface temperature variance in annual cycle

Daytime land surface temperature variance in bi-annual cycle

Daytime land surface temperature variance in tri-annual cycle

Daytime land surface temperature combined variance in annual, bi-annual, and tri-annual cycles

Daytime land surface temperature minimum

Daytime land surface temperature maximum

Daytime land surface temperature phase of annual cycle

Daytime land surface temperature phase of bi-annual cycle

Daytime land surface temperature phase of tri-annual cycle

Daytime land surface temperature variance in raw data

Nighttime land surface temperature mean

Nighttime land surface temperature amplitude of annual cycle

Nighttime land surface temperature amplitude of bi-annual cycle

Nighttime land surface temperature amplitude of tri-annual cycle

Nighttime land surface temperature variance in annual cycle

Nighttime land surface temperature variance in bi-annual cycle

Nighttime land surface temperature variance in tri-annual cycle

Nighttime land surface temperature combined variance in annual, bi-annual, and tri-annual cycles

Nighttime land surface temperature minimum

Nighttime land surface temperature maximum

Nighttime land surface temperature phase of annual cycle

Nighttime land surface temperature phase of bi-annual cycle

Nighttime land surface temperature phase of tri-annual cycle

Nighttime land surface temperature variance in raw data

NDVI (normalized difference vegetation index) mean

NDVI amplitude of annual cycle

NDVI amplitude of bi-annual cycle

NDVI amplitude of tri-annual cycle

NDVI variance in annual cycle

NDVI variance in bi-annual cycle

NDVI variance in tri-annual cycle

NDVI combined variance in annual, bi-annual, and tri-annual cycles

NDVI minimum

NDVI maximum

NDVI phase of annual cycle

NDVI phase of bi-annual cycle

NDVI phase of tri-annual cycle

NDVI variance in raw data

EVI (enhanced vegetation index) mean

EVI amplitude of annual cycle

EVI amplitude of bi-annual cycle

EVI amplitude of tri-annual cycle

EVI variance in annual cycle

EVI variance in bi-annual cycle

EVI variance in tri-annual cycle

EVI combined variance in annual, bi-annual, and tri-annual cycles

EVI minimum

EVI maximum

EVI phase of annual cycle

EVI phase of bi-annual cycle

EVI phase of tri-annual cycle

EVI variance in raw data

Slope from Globe DEM

More information on these data is available at <http://www.edenextdata.com/?q=content/edenext-modis-data>

2) WORLDCLIM (WC), which includes climatic data from 1950 to 2000 [46]:

Average of monthly precipitations of January

Average of monthly precipitations of February

Average of monthly precipitations of March

Average of monthly precipitations of April

Average of monthly precipitations of May

Average of monthly precipitations of June

Average of monthly precipitations of July

Average of monthly precipitations of August

Average of monthly precipitations of September

Average of monthly precipitations of October

Average of monthly precipitations of November

Average of monthly precipitations of December

Average of annual precipitations

Total precipitations

Average of minimum temperature of January

Average of minimum temperature of February

Average of minimum temperature of March

Average of minimum temperature of April

Average of minimum temperature of May

Average of minimum temperature of June

Average of minimum temperature of July

Average of minimum temperature of August

Average of minimum temperature of September

Average of minimum temperature of October

Average of minimum temperature of November

Average of minimum temperature of December

Average of maximum temperature

Average of maximum temperature of January

Average of maximum temperature of February

Average of maximum temperature of March

Average of maximum temperature of April

Average of maximum temperature of May

Average of maximum temperature of June

Average of maximum temperature of July

Average of maximum temperature of August

Average of maximum temperature of September

Average of maximum temperature of October

Average of maximum temperature of November

Average of maximum temperature of December

Average of mean temperature of January

Average of mean temperature of February

Average of mean temperature of March

Average of mean temperature of April

Average of mean temperature of May

Average of mean temperature of June

Average of mean temperature of July

Average of mean temperature of August

Average of mean temperature of September

Average of mean temperature of October

Average of mean temperature of November

Average of mean temperature of December

wc1kprecm e wc1kprect sono precipitazione totale e media da WC

3) a digital elevation model (DEM) created from GLOBE DEM [47].

The 10 variables than together better explicate the predicted variable were selected (table below) with a stepwise inclusive method. Since variables highly correlated with those already present in the tested model are less ameliorative for the model fitness, stepwise inclusive methods tend to select un-correlated variables [49]. To avoid co-linearity the NLDA stepwise selection method use a covariance matrix during the selection process, while the AICc threshold avoids fitting of redundant variables.

As reported in the NLDA average accuracy statistics, the accuracy of the NLDA circulation model, as judged by Cohen’s Kappa (0.8285+/-0.0584), was ‘excellent’ according to Congalton’s classification of kappa values (k<0.4, poor; 0.4<k<0.75, good; and k>0.75, excellent). Model sensitivity (correct presence percentage) and specificity (correct absence percentage) both exceeded 0.90, see table below.

Top 10 variables average rank

Rank Variable

7,400 WC average of mean temperature of August

7,600 ED middle infra-red amplitude of annual cycle

8,000 WC average of mean temperature of February

8,000 WC average of mean temperature of June

8,100 ED middle infra-red variance

8,500 ED EVI amplitude in tri-annual cycle

8,600 ED daytime LST amplitude of tri-annual cycle

8,700 ED daytime LST minimum

8,800 ED NDVI amplitude of bi-annual cycle

9,000 ED NDVI variance

Average NLDA accuracy statistics:

Kappa: 0.8285 +/- 0.0584

AUC: 0.9693 +/- 0.0317

Sensitivity: 0.9015 +/- 0.0402

Specificity: 0.9569 +/- 0.0306
