# Supplementary material for: West Nile Virus Surveillance in 2013 via Mosquito Screening in Northern Italy and the Influence of Weather on Virus Circulation
Source: PLoS One. 2015 Oct 21;10(10):e0140915. doi: 10.1371/journal.pone.0140915 (PMC4619062; doi:10.1371/journal.pone.0140915)
Supplement: S5 Table — (DOCX) [file pone.0140915.s008.docx]

**S5 Table. Identity between the consensus of USUV field detected sequences and other homologous sequences.** Number of base differences (lower left) and percentage of identity (upper right) between the consensus of USUV field detected sequences and other homologous sequences deposed in Gen Bank (GB). Alignment of 200 base pairs of the NS5 gene, * sequences detected in the surveyed area.

|  | Strain | Country | Year | Origin | GB | 1 | 2 | 3 | 4 | 5 | 6 | 7 | 8 | 9 | 10 | 11 | 12 |
| --- | --- | --- | --- | --- | --- | --- | --- | --- | --- | --- | --- | --- | --- | --- | --- | --- | --- |
| 1 | This study | Italy | 2013 | Mosquito | - |  | 100 | 99.5 | 99.0 | 99.0 | 98.5 | 98.0 | 97.5 | 97.5 | 97.5 | 97.0 | 96.5 |
| 2 | USU-RE-m7/2010 | Italy* | 2010 | Mosquito | JF834591 | 0 |  | 99.5 | 99.5 | 99.5 | 99.0 | 98.5 | 98.0 | 98.0 | 98.0 | 97.0 | 96.5 |
| 3 | Bologna 2009 | Italy* | 2009 | Human | HM569263 | 1 | 1 |  | 99.0 | 99.0 | 98.5 | 98.0 | 97.5 | 97.5 | 97.5 | 97.0 | 96.5 |
| 4 | Vienna_2001 | Vienna | 2001 | Bird | AY453411 | 2 | 1 | 2 |  | 100 | 98.5 | 99.0 | 98.5 | 97.5 | 98.5 | 96.5 | 96.0 |
| 5 | Meise H | Austria | 2002 | Bird | JQ219843 | 2 | 1 | 2 | 0 |  | 98.5 | 99.0 | 98.5 | 97.5 | 98.5 | 96.5 | 96.0 |
| 6 | m2080-NO/ITA/2011 | Italy* | 2011 | Mosquito | KF882514 | 3 | 2 | 3 | 3 | 3 |  | 98.5 | 98.0 | 98.0 | 98.0 | 97.0 | 96.5 |
| 7 | Milan 2006 | Italy* | 2006 | Bird | JX473242 | 4 | 3 | 4 | 2 | 2 | 3 |  | 98.5 | 97.5 | 98.5 | 96.5 | 96.0 |
| 8 | BAT2USUTU-BNI | Germany | 2013 | Bat | KJ859683 | 5 | 4 | 5 | 3 | 3 | 4 | 3 |  | 97.0 | 100 | 96.0 | 96.5 |
| 9 | ArD192495 | Senegal | 2007 | Mosquito | KC754957 | 5 | 4 | 5 | 5 | 5 | 4 | 5 | 6 |  | 97.0 | 99.0 | 97.5 |
| 10 | BH65/11-02-03 | Germany | 2011 | Bird | HE599647 | 5 | 4 | 5 | 3 | 3 | 4 | 3 | 0 | 6 |  | 96.0 | 96.5 |
| 11 | HB81P08 | C. African Rep. | 1981 | Human | KC754955 | 6 | 6 | 6 | 7 | 7 | 6 | 7 | 8 | 2 | 8 |  | 97.5 |
| 12 | SAAR-1776 | South Africa | 1959 | Mosquito | AY453412 | 7 | 7 | 7 | 8 | 8 | 7 | 8 | 7 | 5 | 7 | 5 |  |
